# Barriers to growth hormone access in pediatric patients at an academic medical center

**DOI:** 10.1093/ajhp/zxaf232

**Published:** 2025-09-02

**Authors:** Chardé Parrish, Danielle Baird, Terra Redd, Ryan P Moore, Leena Choi, Autumn D Zuckerman, Katie R Cruchelow

**Affiliations:** Vanderbilt Specialty Pharmacy, Vanderbilt Health System, Nashville, TN, USA; Vanderbilt Specialty Pharmacy, Vanderbilt Health System, Nashville, TN, USA; College of Pharmacy, Lipscomb University, Nashville, TN, USA; Department of Biostatistics, Vanderbilt University Medical Center, Nashville, TN, USA; Department of Biostatistics, Vanderbilt University Medical Center, Nashville, TN, USA; Vanderbilt Specialty Pharmacy, Vanderbilt Health System, Nashville, TN, USA; Vanderbilt Specialty Pharmacy, Vanderbilt Health System, Nashville, TN, USA

**Keywords:** growth hormone, growth hormone therapy, medication access, prior authorization

## Abstract

**Purpose:**

Evaluating barriers that may delay or limit human growth hormone (hGH) access is important to ensure timely and equitable treatment. We examined time to hGH access and whether time to approval was associated with patient factors and impacts on patient growth.

**Methods:**

This was a single-center, retrospective review of the electronic medical records of pediatric patients starting hGH treatment for an indication approved by the FDA at the Vanderbilt Pediatric Endocrinology Clinic between January 1, 2018, and December 31, 2020. A Cox proportional hazards model was used to assess factors associated with time to access. Linear regression analysis was used to assess factors impacting patients’ change in height *z* score from baseline to 1 year after the decision to treat (DTT).

**Results:**

The inclusion criteria were met by 374 patients. Patients had a median age of 11 years (interquartile range [IQR], 8-13 years), 66% were male, 80% identified as White, and the median pretreatment height *z* score was –2.5 (IQR, –3 to –2). The median time to access was 3 days (IQR, 1-6 days) with a prior authorization, 34 days (IQR, 22-68 days) with an appeal, 84 days (IQR, 59-122 days) with a patient assistance program, and 94 days (IQR, 46-155 days) with cash pay. Diagnosis (*P* = 0.002), additional testing (*P* < 0.0001), and method of final access (*P* < 0.001) were significantly associated with the time to access. Height *z* score at DTT (*P* < 0.001), diagnosis (*P* = 0.021), and race (*P* = 0.045) were significantly associated with the change in height at 1 year after DTT.

**Conclusion:**

Diagnosis, insurance, additional testing, and method of medication approval were associated with time to access. Time to hGH initiation did not impact patient growth 1 year after DTT.

Key Points

- Access to growth hormone can be difficult to obtain due to costs and the insurance authorization process. Understanding factors that affect access can help address barriers and promote pharmacoequity.
- The majority of patients (71%) were approved on prior authorization. The remaining 29% required an appeal, enrolled in a patient assistance program through the manufacturer, or paid out of pocket, leading to a median delay of 3 months from the decision to treat.
- Patient diagnosis, insurance type, additional testing required by insurance, and method of approval were associated with time to approval.

The introduction of recombinant human growth hormone (hGH) in 1985 has expanded the scope of practice for growth hormone therapy in pediatric endocrinology.^1^ Therapy that was once used strictly for replacement of growth hormone deficiency (GHD) now has many approved indications from the Food and Drug Administration (FDA) for pharmacological hormonal augmentation, such as idiopathic short stature, small for gestational age, Noonan syndrome, Prader-Willi syndrome, Turner syndrome, and chronic kidney disease (CKD). Interest in improving height for children who are short for reasons other than GHD has increased, recognizing that severe short stature can be a disabling condition affecting a child’s physical and psychological well-being. The expanded use of hGH therapy is supported by its favorable safety profile, even at escalating dosages, and its ability to significantly enhance quality of life.^1^

Beyond promotion of linear growth, hGH plays a crucial role in various physiological processes, including metabolic regulation, enhancement of muscle strength, improvement of bone mineral density, and modulation of lipid profiles. By contributing to overall growth and development, growth hormone affects organs and tissues throughout the body, ultimately leading to better health outcomes.^2^

Access to growth hormone can be difficult to obtain due to costs and the insurance authorization process that often involves more stringent requirements beyond having an FDA-approved indication. Studies have suggested that replacement of growth hormone at younger ages results in faster and greater growth; therefore, time to approval and time to access hGH are important variables in the care of patients prescribed hGH. Because there is limited time for growth before epiphyses close, early initiation of hGH is imperative.^3^

However, navigating access to care may be a challenge for patients and caregivers for many reasons. Patients needing hGH must often be seen by pediatric endocrinologists, which requires a referral from a pediatrician. Perceived standards of acceptable height and the urgency to seek treatment may vary among patient groups due to differences in education levels, cultural norms, or comorbidities.^4^ Explicit and implicit biases among providers may impact referral patterns. Once treatment with hGH is prescribed by a pediatric endocrinologist, patient access to medication can be delayed or impeded for several reasons. Growth hormone is a high-cost specialty medication that requires prior authorization (PA) from the insurance provider. If the PA request is denied, appeal(s) and sometimes external reviews are required to obtain patient access to medication. Yet, even with insurance approval, some patients are unable to afford medication copays without financial assistance. If insurance approval cannot be obtained, some patients may qualify for patient assistance from the drug manufacturer, which can also require a lengthy approval process.

Understanding factors that affect access can help address barriers and promote pharmacoequity.^5^ The literature evaluating patient and medication-related factors that affect growth is also limited. The purpose of this study was to examine the time to hGH insurance approval, determine factors impacting time to access, and evaluate factors impacting growth after a decision to treat (DTT) with hGH.

## Methods

### Patients

This was a single-center, retrospective review of the electronic medical records of pediatric patients starting hGH treatment (Norditropin, Nutropin, Genotropin, Humatrope, Omnitrope, Saizen, or Zomacton) for am FDA-approved indication (idiopathic short stature, GHD, panhypopituitarism, Turner syndrome, small for gestational age, or Prader-Willi syndrome) between January 1, 2018, and December 31, 2020. Patients were excluded if the DTT was rescinded before prescription approval, the prescription was an hGH renewal, there was a change in hGH due to insurance changes, the patient chose to pay out of pocket before starting the insurance approval process, or there was a diagnosis of adult hGH deficiency.

### Setting

**Figure 1.**
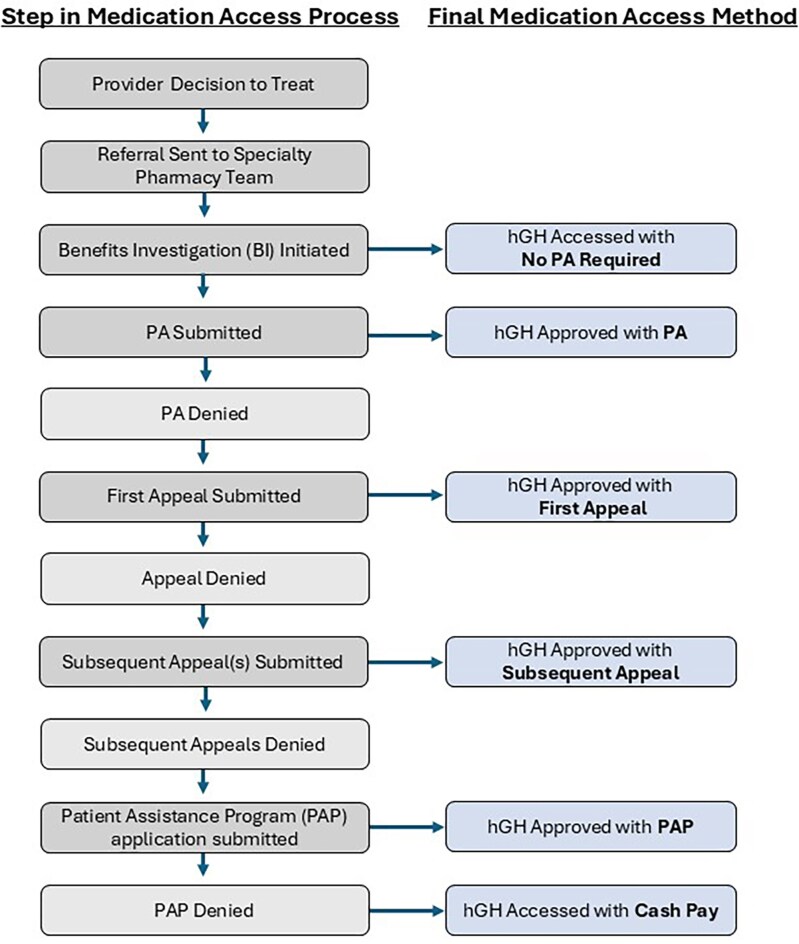
Medication access process. BI indicates benefits investigation; hGH, human growth hormone; PA, prior authorization; PAP, patient assistance program.

This study was performed at a single academic medical center in the southeastern US with an integrated health-system specialty pharmacy (IHSSP). Two specialty pharmacists and 2 pharmacy technicians are integrated into an outpatient pediatric endocrinology clinic, working alongside clinic staff communicating in the electronic health record (EHR). When a provider makes the decision to treat a patient with hGH, a referral is sent to the specialty pharmacist integrated into the pediatric endocrinology clinic. The specialty pharmacist starts the insurance approval process by performing a benefits investigation to assess the patient’s insurance status and medication access requirements. A PA is not always required for access. If needed, a PA request is submitted by the pharmacy technician with the patient’s insurance company. If the PA is denied, the pharmacist writes an appeal letter detailing the patient’s need for the hGH medication, providing clinical data and primary literature to support their case. If the appeal is denied, the pharmacy team writes a second appeal letter providing additional information or works with the provider to complete a peer-to-peer review, depending on the insurance requirements. If a second or third appeal is denied, the pharmacist discusses other treatment options with the provider. If a patient’s insurance continues to deny access to the medication, the pharmacy team works with the patient on gaining medication access through patient assistant programs (PAPs) to cover the cost of the medication, if certain requirements are met. However, not all patients will be approved for PAPs, leaving them with the final option of paying the entire medication cost out of pocket (ie, cash pay). Once access to the medication is approved, a new prescription is generated and sent to the patient’s pharmacy (Figure 1).

### Data collection

Data for patients prescribed hGH during the study period were extracted from the EHR. Data extracted included dates for prescriptions, benefits investigation outcomes, PA and appeals, PAP enrollment, and cash pay. The patient’s height closest to the DTT date was also extracted from the EHR. Additional data gathered included the diagnosis, the reason for PA denials, interim medications (if applicable), the patient’s filling pharmacy, and additional tests needed per insurance requirements. Data were stored in REDCap, hosted at Vanderbilt University Medical Center.^6,7^ This study was approved by the institutional review board.

### Outcomes

The primary outcome was the time to hGH access, defined as the number of days between the prescribing provider’s treatment decision and final medication access (insurance approval, PAP enrollment, or cash pay). Medication access could occur without the need for a PA, after a PA was completed, or after up to 3 appeals. Patients could be approved through a PAP if insurance denied access after exhausting all appeal options. Finally, if the patient was never approved by insurance and did not qualify for a PAP, they could elect to pay the full cost for treatment out of pocket.

The secondary outcome was height at 1 year after DTT. Instead of using raw height data, height *z* scores were used to adjust for age and sex. Patients had multiple height measurements spanning from before DTT to beyond 1 year after DTT, but measurements were generally not available precisely at DTT or exactly 1 year later. To address this, individual regression lines were fit for each patient and predicted heights at DTT and 1 year after DTT were used to define baseline height and the outcome of height at 1 year after DTT, respectively.

### Statistical analysis

Patient characteristics and time to access were summarized using the median and interquartile range (IQR) for continuous variables and count and proportion for categorical variables. A Cox proportional hazards model was used to assess factors associated with time to access. The factors we assessed included the height *z* score at DTT, diagnosis, insurance type, additional testing required, method of approval, and pharmacy filling the prescription. Linear regression analysis was used to assess factors impacting patients’ change in height *z* score from baseline to 1 year after DTT, including the baseline predicted height *z* score at DTT, race, time to approval, interim medications used, diagnosis, additional testing required, and pharmacy filling the prescription. Height *z* scores were calculated using World Health Organization (WHO) and Centers for Disease Control and Prevention (CDC) growth standards to adjust for age and sex. The WHO and CDC growth standards are available for different age ranges, so a combination of the scores was used. WHO growth standards were used for patients less than 24 months old, and CDC standards were used for patients who were 24 months of age or older. Analyses were conducted with the R programming language (Version 4.4.18).^8^

## Results

The inclusion criteria were met by 374 patients. Patients had a median age of 11 years (IQR, 8-13 years), 66% were male, 80% identified as White, and the median pretreatment height *z* score was –2.5 (IQR, –3 to –2). The most common diagnosis was GHD (49%), followed by idiopathic short stature (30%) (Table 1).

**Table 1.** Characteristics of the Sample

Characteristic^a^	Patients (N = 374)
Age, median (IQR), years	11.2 (8.1-13.3)
Gender	
Female	126 (34)
Male	248 (66)
Race	
White	301 (80)
Black	20 (5.3)
Other	29 (7.8)
Unknown	24 (6.4)
Ethnicity	
Not Hispanic, Latino/a, or Spanish origin	158 (42)
Mexican, Mexican American, or Chicano/a	5 (1.3)
Other Hispanic, Latino/a, or Spanish origin	32 (8.6)
Cuban	1 (0.3)
Unknown	167 (45)
Decline to answer	11 (2.9)
Language	
English	353 (94)
Non-English	21 (5.6)
Baseline height *z* score (WHO or CDC), median (IQR)	–2.5 (–3 to –2)
Diagnosis	
Chronic kidney disease	5 (1.3)
Growth hormone deficiency	185 (49)
Idiopathic short stature	113 (30)
Noonan syndrome	1 (0.3)
Panhypopituitarism	7 (1.9)
Prader-Willi syndrome	11 (2.9)
Small for gestational age	35 (9.4)
Turner syndrome	17 (4.5)
Primary insurance	
Commercial	245 (66)
Medicaid	117 (31)
Tricare	12 (3.2)
Filling pharmacy	
HSSP	193 (52)
Non-HSSP	181 (48)

Abbreviations: CDC, Centers for Disease Control and Prevention; HSSP, health-system specialty pharmacy; IQR, interquartile range; WHO, World Health Organization.

^a^Data shown as No. (%) unless indicated otherwise.

**Figure 2.**
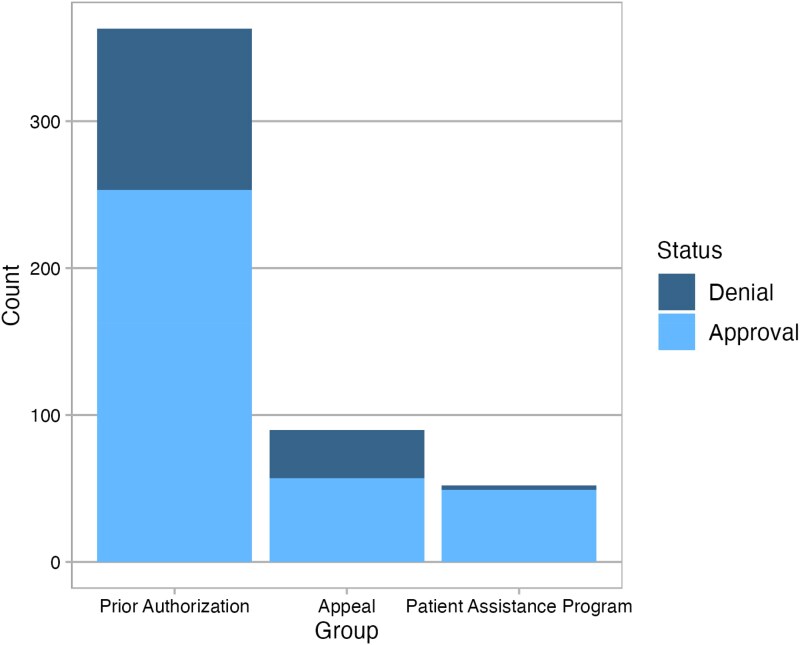
Method of final medication access.

**Figure 3.**
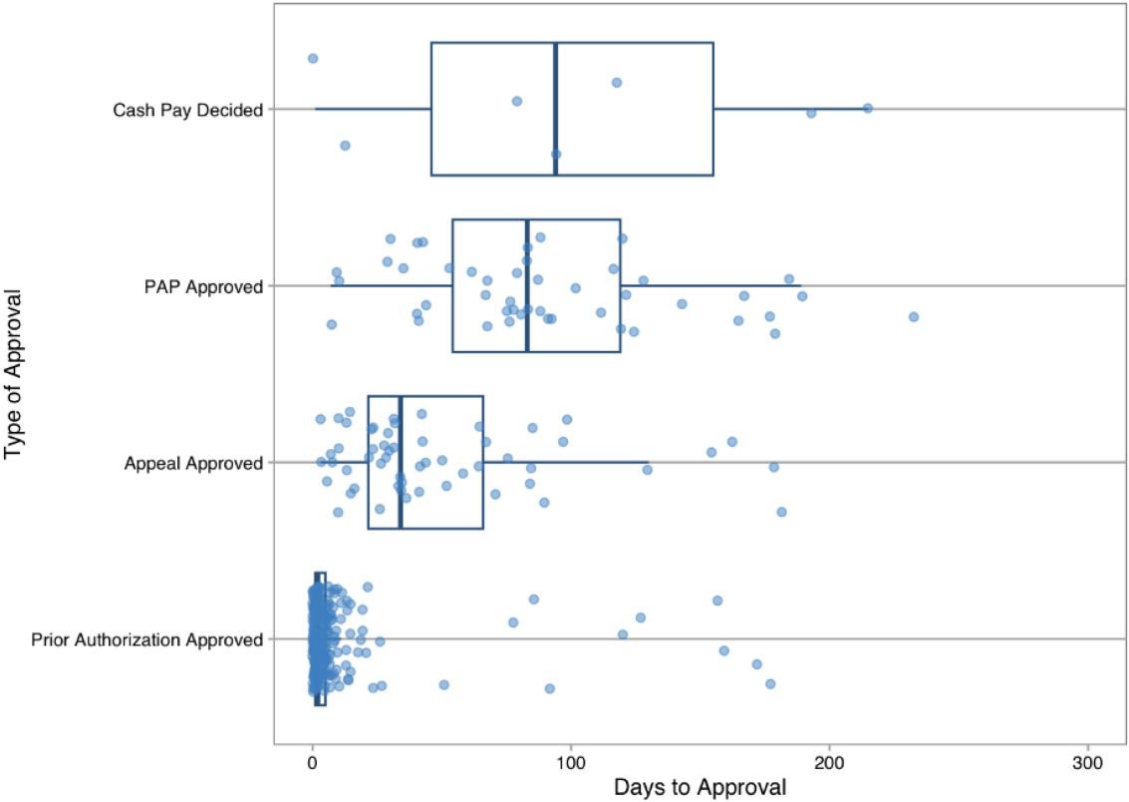
Time from decision to treat to medication access, by access method. PAP indicates patient assistance program.

Of the 374 patients, 71% were approved after the initial PA request, 15% required an appeal, 12% utilized a PAP, and 2% decided to use cash pay after going through the insurance approval process (Figure 2). The median time from DTT to medication access was 3 days (IQR, 1-6 days) in patients needing an insurance PA, 34 days (IQR, 22-68 days) if an appeal was required, 84 days (IQR, 59-122 days) if the patient was not approved through insurance and used a manufacturer’s PAP, and 94 days (IQR, 46-155 days) if the patient was not approved through insurance and ultimately paid out of pocket (Figure 3).

**Figure 4.**
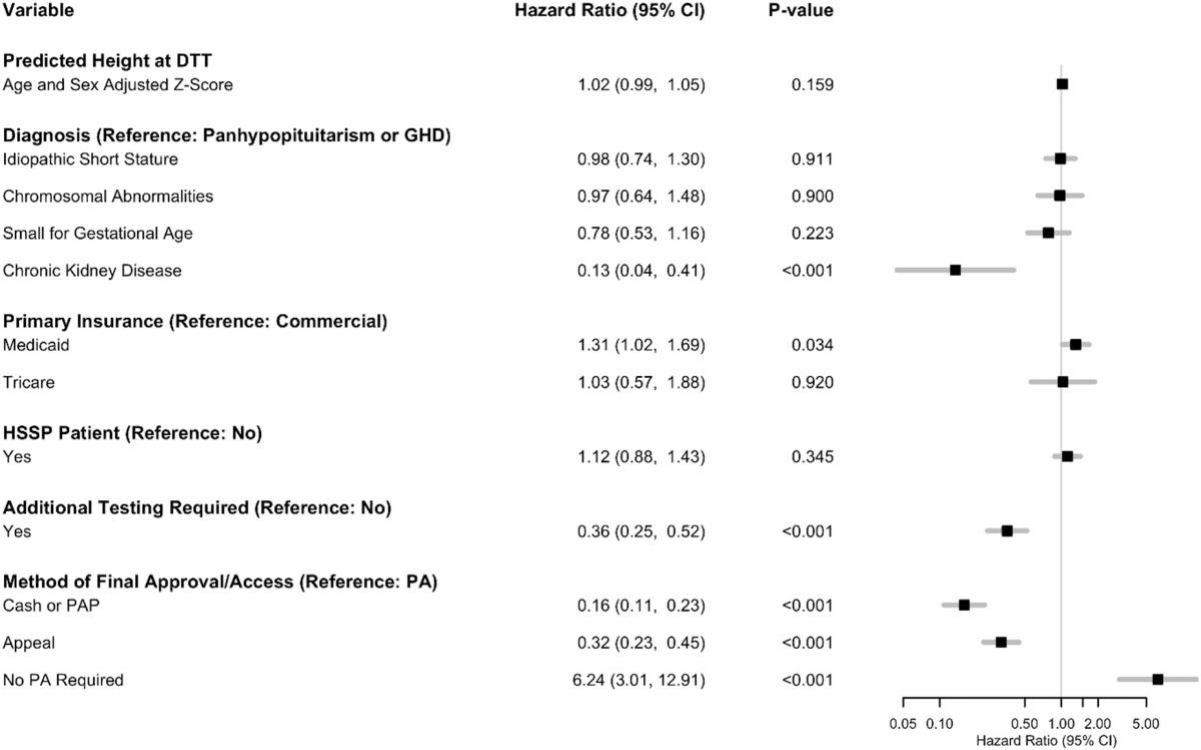
Cox proportional hazards regression to examine factors associated with time to approval. The gray line in the forest plot indicates a hazard ratio of 1. CI indicates confidence interval; DTT, decision to treat; GHD, growth hormone deficiency; HSSP, health-system specialty pharmacy; PA, prior authorization; PAP, patient assistance program.

We performed Cox proportional hazards regression to examine factors associated with time to access. The model included diagnosis, predicted height *z* score at DTT, insurance type, filling pharmacy, requirement for additional testing, and method of final coverage as independent variables. Diagnosis was significantly associated with time to access (*P* = 0.002). Patients whose insurance required additional testing had significantly longer times to access (hazard ratio [HR], 0.36; 95% confidence interval [CI], 0.25-0.52; *P* < 0.001). Additionally, patients with Medicaid had a significantly shorter time to access than those with commercial insurance (HR, 1.31; 95% CI, 1.02-1.69; *P* = 0.034). The method of approval was also significantly associated with time to access (*P* < 0.001). Each additional step in the medication coverage process was associated with longer time to access. Compared to patients who required PA approval, those who did not require a PA had significantly shorter times to access (HR, 6.24; 95% CI, 3.01-12.91; *P* < 0.001). However, patients whose insurance required an appeal for access and those who were never approved but received medication coverage through a PAP or paid out of pocket had significantly longer times to access (HR, 0.32; 95% CI, 0.23-0.45; *P* < 0.001 and HR, 0.16; 95% CI, 0.11-0.23; *P* < 0.001, respectively) (Figure 4).

We conducted linear regression analysis to identify factors associated with the change in height *z* score from baseline to 1 year after DTT. The baseline height *z* score at DTT was negatively associated with the change in height *z* score at 1 year after DTT, with taller patients having a smaller expected amount of growth (coefficient, –0.08; 95% CI, –0.11 to –0.04; *P* < 0.001). Race also had a significant association with the change in height *z* score (*P* = 0.045), with Black patients having a lower change in height relative to White patients (coefficient, –0.16; 95% CI, –0.28 to –0.03; *P* = 0.015). Diagnosis was associated with the change in height *z* score (*P* = 0.021), with patients with idiopathic short stature and small for gestational age having a lower change in height *z* score relative to patients with panhypopituitarism and GHD (coefficient, –0.09; 95% CI, –0.16 to –0.02; *P* = 0.010 and coefficient, –0.11; 95% CI, –0.21 to –0.01; *P* = 0.037, respectively) (eFigure 1).

## Discussion

Study findings demonstrated that access to growth hormone can be a long and tedious process for many patients. Almost 30% of patients required an appeal, enrolled in a PAP through the manufacturer, or paid out of pocket, leading to a median delay of 3 months from the DTT. Most patients began treatment within 3 months, but time to access was influenced by factors such as insurance type, diagnosis, and insurer-mandated testing.

### Time to access

The median time to hGH access was 3 days, highlighting how the IHSSP model helps patients efficiently access and initiate therapy. IHSSP pharmacy technicians and specialty pharmacists use the patient’s EHR to accurately and comprehensively complete insurance requirements.^9^ Patients were frequently denied insurance coverage for hGH therapy due to their diagnosis not meeting formulary criteria. The diagnosis of idiopathic short stature is excluded from several insurance plans, despite being an FDA-labeled use indication for growth hormone. Multiple studies have shown the benefit of hGH for these patients.^10,11^ Patients with idiopathic short stature have used manufacturer assistance programs in the past to gain access, but, unfortunately, several manufacturers have closed their PAPs, reducing access to hGH therapy. This has caused hGH to be inaccessible for many patients. Although an indication of CKD was associated with longer time to approval relative to panhypopituitarism or GHD, this was likely due to few patients in the current study being prescribed hGH for CKD (n = 5).

Patients whose insurance required additional testing were less than half as likely to gain access at any given time relative to those whose insurance did not require such testing. Insurance requirements for testing before hGH initiation in a patient with a diagnosis of GHD vary based on the insurance. For plans that require testing, multiple tests and values may be required, including growth hormone stimulation testing, insulin-like growth factor 1 levels, bone age reports, height standard deviation limits, and brain magnetic resonance imaging. These tests are expensive and time consuming for young children and their caregivers. Additional testing increases healthcare costs overall, which can place a burden on those with health and social disparities.

Endocrinologists are often keenly aware of the clinical testing required for their patients. However, the variability in insurance requirements makes it challenging to ensure that all necessary documentation is submitted at the outset, often leading to delays. Transparency and consistency in insurance requirements could allow providers to plan more effectively, reducing the need for patients and caregivers to return for additional testing or documentation. Although proactive planning may not eliminate all follow-up visits before initiating hGH, it could help streamline the access process and minimize delays for families. The utility of requiring additional testing, considering the burden it places on patients, caregivers, and the healthcare system, warrants further scrutiny. For vulnerable populations, these challenges may exacerbate existing disparities in access to timely and appropriate care.

### Differences in change in height from DTT to 1 year after DTT

This study did not find that time to access made a difference in patients’ change in height 1 year after DTT. However, most of our patients were approved within 3 months. On the basis of the literature, age at growth hormone initiation is likely the most important factor in improving height.^3^ In the current study, taller patients at baseline had less expected growth through 1 year after DTT. Existing literature also suggests that children with shorter baseline heights tend to experience more significant growth during hGH therapy. Research from the ANSWER Program highlighted the importance of baseline height in predicting treatment response, with children starting at shorter heights showing greater gains over time.^12^ These findings illustrate the importance of identifying and treating children as early as possible to optimize growth outcomes.

Patients with idiopathic short stature and those small for their gestational age were predicted to have lower changes in height *z* score at 1 year compared to patients with panhypopituitarism or GHD.^13^ Other studies have also shown that the response to growth hormone of patients with idiopathic short stature may be less than that in patients with other conditions and that their response can be highly variable. However, overall, growth hormone is still indicated for idiopathic short stature and has been shown to increase adult height in these patients.

Our finding of race as a significant factor associated with height at 1 year after DTT aligns with previous research, which indicated that Black patients initiating hGH therapy present with greater height deficits and lower peak levels compared to White patients. Many factors can contribute to the difference in growth for Black patients compared to White patients, such as genetic and socioeconomic factors.^14,15^ Previous research has shown that racial disparities are seen for children with short stature specifically.^16^ Additionally, evidence suggests that Black children who are referred for short stature are shorter during growth hormone stimulation tests and have lower peak levels compared to their White counterparts.^14^ While this initially suggests racial differences, it is important to recognize that these greater deficits in Black patients may reflect healthcare disparities. Black patients may not receive timely referrals for treatment and may therefore present with more severe growth impairment by the time treatment is initiated.^15,17^ For a child to be evaluated for GHD, their caregivers’ attitudes and levels of concern for growth make an impact on whether a child is referred to a specialty clinic. In retrospective studies, Black families have been reported to think that height is a minor problem (relative to more important issues), leading to a higher threshold to consider hGH deficiencies, especially short stature, an issue.^1,18^ Overrepresentation of White children in hGH treatment registries points to potential systemic biases in access to care, which could influence overall treatment outcomes by race.

### Limitations

Because most patients were able to access hGH within 3 months, there was a small sample of patients with long delays in access. A larger sample of patients with prolonged therapy delays might have allowed us to detect differences in height outcomes. Growth may be influenced by factors not fully accounted for in this study, such as patient adherence, nutritional status, or concurrent medical conditions. Each of these factors plays a crucial role in growth and can be difficult to monitor or quantify. Additional patient factors such as missed appointments, hGH regimen, and dose adjustments were not collected in the current study but should be included in the future to examine additional factors that could affect patient growth. Our findings might not be applicable to all races, socioeconomic backgrounds, or geographic regions. Healthcare disparities can also play a large role in the outcomes of patient care. In addition to the factors evaluated in the current study, access to growth hormone has become more complicated due to changes to many manufacturer PAPs limiting financial assistance options. The ongoing growth hormone shortage, which began in January 2023, significantly delayed therapy onset as patients often required multiple PAs or appeals due to nonpreferred agents being the only available options. However, as of summer 2024, the shortage appeared to be largely resolved, although the impact on patient care during the shortage was substantial. Initiating treatment early can affect overall growth, with early initiation leading to higher adult heights. Therefore, addressing inequities in growth hormone access is essential to minimize delays and optimize patient outcomes.

## Conclusion

Access to hGH therapy is hindered by the number of steps required to obtain medication coverage and varying insurance requirements for additional testing, which may place undue burden on populations already facing challenges in accessing care. To promote pharmacoequity and ensure hGH access for all patients in need, it is essential to implement standardized insurance requirements that align with clinical guidelines and minimize patient burden. Study findings also highlight the critical role of noninsurance coverage options such as manufacturer PAPs and cash pay in enabling many patients to obtain hGH treatment. Because taller patients who started hGH treatment had less expected growth, prioritizing early diagnosis and intervention may maximize treatment benefits. Additionally, given the impact of diagnosis and race on growth outcomes, the study highlights the need to further evaluate and address potential disparities in access to hGH therapy. By addressing challenges in access to care, we can improve hGH access and outcomes, ultimately promoting health equity for all patients in need.

## Disclosures

Dr. Zuckerman reports research support with funding going to the institution from Sanofi, AstraZeneca, Pfizer, BeiGene, and UCB, unrelated to this study. Dr. Cruchelow reports research support with funding going to the institution from Sanofi and UCB, unrelated to this study. All other authors have declared no potential conflicts of interest.

## Supplementary Material

zxaf232_Supplementary_Data
