# Supplementary material for: Barriers to growth hormone access in pediatric patients at an academic medical center
Source: Am J Health Syst Pharm. 2025 Sep 2;83(6):272–80. doi: 10.1093/ajhp/zxaf232 (PMC13016747; doi:10.1093/ajhp/zxaf232)

**eFigure 1.** Linear regression analysis to identify factors associated with the change in height Z-score at one year post DTT. The grey line in the forest plot indicates a coefficient of 0.


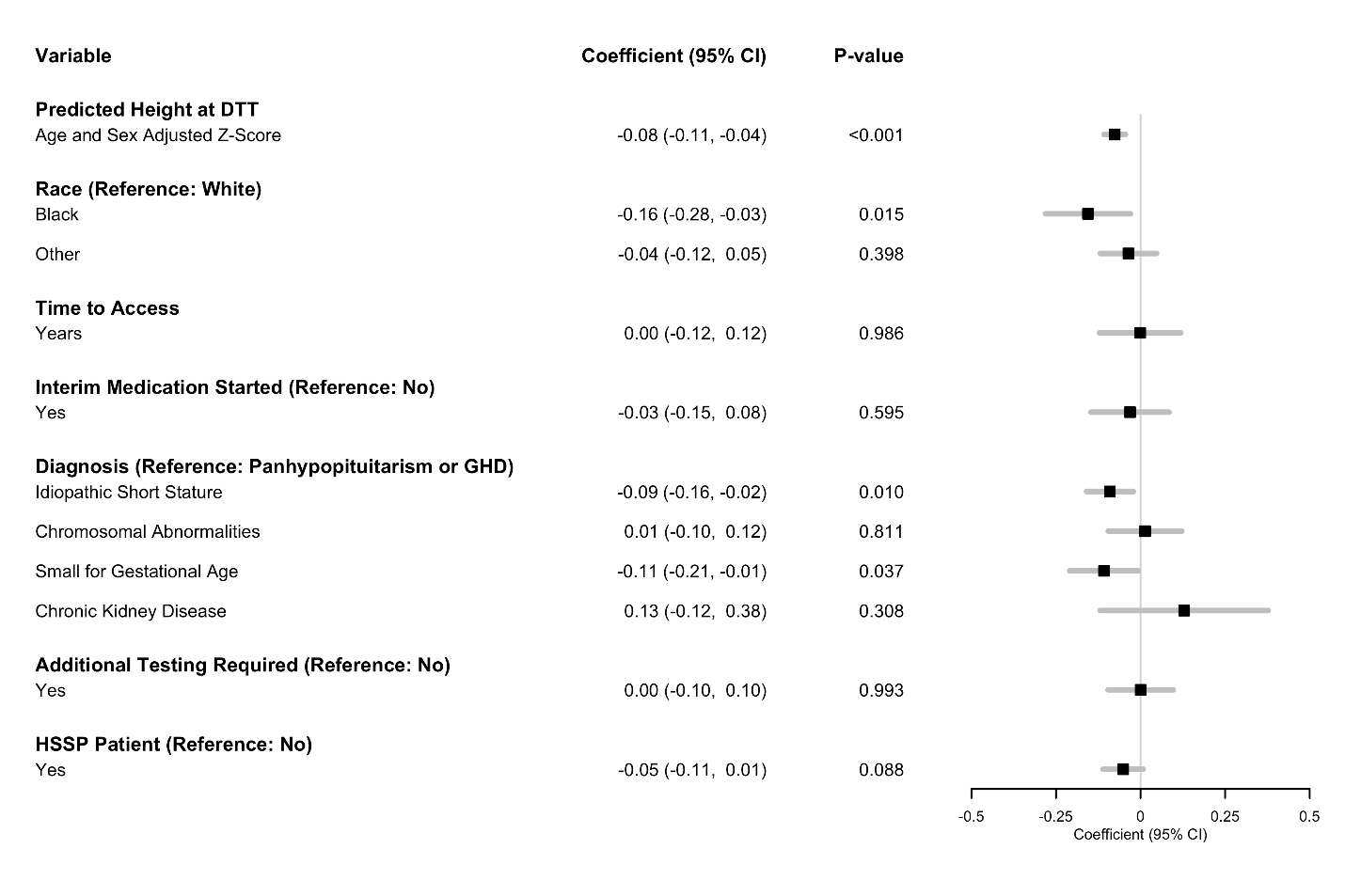

Supplement: zxaf232_Supplementary_Data [file zxaf232_supplementary_data.docx]
